# Networked Learning: Inviting Redefinition

**DOI:** 10.1007/s42438-020-00167-8

**Published:** 2020-07-22

**Authors:** Networked Learning Editorial Collective (NLEC)

**Affiliations:** Lancaster University, Lancaster, UK

**Keywords:** Networked learning, Learning networks, Collaborative learning, Collaborative inquiry, Socio-material, Design, Agency, Emancipatory education, Postdigital

## From Emergency Remote Teaching to Networked Learning

In the opening months of 2020, educational institutions in many countries showed themselves capable of making dramatic changes in their ways of working. These moves were occasioned by the Covid-19 pandemic, though the actual responses of institutions were shaped by quite diverse factors. A common strategy was to use digital communications technologies to continue with some fundamental elements of educational provision. In the higher education sector, commentators and university leaders began to refer to this as a ‘pivot’ to ‘online learning’.

Hodges, Moore, Lockee, Trust, and Bond (2020), Czerniewicz (2020) and others have reminded us of the importance of language in large-scale shifts of this kind. For example, reviews of research showing that online learning can produce academic outcomes equivalent to face-to-face or ‘on campus’ teaching may be used to quieten students’ concerns about the quality of their changed educational experiences. Such arguments, while often well-intentioned, use a sleight-of-hand that equates professionally designed online programs with rapidly improvised forms of teaching. Less well-intentioned arguments may be used to propose ongoing cuts to resources or the ‘unbundling’ of university services to allow profitable parts of university education to be taken over by commercial providers, through the monetised successors to Massive Open Online Courses (MOOCs), for example (Selwyn 2020).

So language choices matter. As universities plan their ways forward, *how* they describe past, current and future arrangements may have significant consequences. Hodges et al. (2020) suggest the term ‘emergency remote teaching’ for the improvised arrangements that were quickly set in place in the first half of 2020. But how should we describe more planful arrangements, going forward?

‘Online learning’ has always been an awkward term—not least, because, like ‘digital’, ‘distance’ and ‘virtual’, it can obscure the embodied and physically situated nature of learning (Fawns 2019). Students live in a complex social-material-digital world and the learning places they make affect how they learn. There is a small body of good research on how so-called ‘online’ and ‘distance’ students make places to study—at home, at work and elsewhere (e.g. Jaldemark 2008; Jones and Healing 2010; Bayne, Gallagher, and Lamb 2013; Gourlay and Oliver 2018). The Covid-19 ‘lockdown’ has been generating anecdotes and further insights into the effects of home-based learning places on opportunities for study.

University leaders who are planning the staged re-opening of campuses are picking up the language of ‘blended learning’ to indicate how the future pattern of educational provision will involve a mixture of home-based ‘online’ and campus-based ‘face-to-face’ provision. In crude terms, the former will deal with ‘theory’ and ‘content’ and the latter with ‘hands-on skills’ (science lab classes, engineering workshops, clinics, etc). Although there is a literature on ‘blended’ learning, the term is slippery to define (Oliver & Trigwell, 2005; Bliuc, Ellis, and Goodyear 2007; Hrastinski 2019). For some meta-analyses and research reviews, it has been operationalized in such a stark way that the findings bear little relationship to contemporary educational practice (Means, Toyama, Murphy, and Baki 2013; Bernard, Borokhovski, Schmid, Tamim, and Abrami 2014).

Many university students in richer countries now carry laptops and mobile phones. Well-resourced students can view lectures at times and in places that suit them (lockdown regulations allowing). They form their own self-supporting study groups on social media. In normal times, many on-campus activities also involve digital tools and resources. As Bill Mitchell forecast, we have seen an accelerating interpenetration of the digital and material, spaces have become hybrid and digital infrastructures are taken for granted (Mitchell 2003; Guribye 2015; Goodyear 2020). Even in countries with fewer resources and strong digital divides, inventive practices using mobile phones are creating rich meshworks of learning relationships (Czerniewicz and Brown 2012; Timmis and Muhuro 2019). It is now rare to find real learning situations that can be described as ‘purely face-to-face’ or ‘wholly online’. Rather, they involve complex entanglements of students, teachers, ideas, tasks, activities, tools, artefacts, places and spaces (Carvalho and Yeoman 2018; Ryberg, Davidsen and Hodgson 2018).

There is a field of research and practice in education that studies such entanglements. It is known as networked learning. Over the last 20 years or so, researchers in this field have developed methods for analysing learning networks and designing for networked learning.

In late May 2020, the networked learning community held its 12th biennial international conference. One of the roundtable sessions at the conference revisited the definition of ‘networked learning’ to consider whether it was still fit for purpose or in need of updating. An early draft of this paper was made available to delegates at the conference. In this published form, the paper aims to promote discussion of a revised definition of ‘networked learning’. This process is intended to increase the visibility of an aspect of networked learning that has long been a strength but which was not reflected in its customary definition. It recognizes networked learning’s roots in critical and emancipatory educational traditions. It underscores a commitment to equity and social justice and invites contributions to the challenges of transitioning to more sustainable forms of living. In our view networked learning has a great deal to offer to teachers and leaders who want a distinctive label for a more ambitious conception of education, moving forward (Peters et al. 2020).

## What Is Special About Networked Learning?

Networked learning is distinguished as a field of research and practice by its insistent attention to three sets of phenomena and to their intertwinement in practice:

- Human/inter-personal relationships
- Technology (especially digital communications technologies)
- Collaborative engagement in valued activity (joint inquiry, knowledgeable action, etc).

While the focus for a specific research study or educational design enhancement may temporarily shift to just one of these three, the other two can never be ignored. So, for example, new digital platforms and devices become interesting in relation to human activities and social practices.

- An insistence on the importance of *human relationships* opens up questions about trust, power, identity, belonging, difference, affection, reciprocity, solidarity, commitment and time.
- An interest in how *technologies* shape and are shaped by human activity, with a recognition that tools, artefacts and infrastructure are assembled or reconfigured in complex ways, provokes questions about the socio-material, affordances, instruments, access, appropriation, ownership, etc.
- A commitment to *collaborative inquiry and joint action in the face of shared challenges* raises questions about knowledge, values and action, learning and doing, meaning-making, negotiation, shared projects and praxis, scale, scope, pace and duration and the capabilities needed to shape a world worth living in.

These are far from being exhaustive. Moreover, attention to the binary and ternary relations between these three strands raises further sets of questions, opportunities and constraints for research and design.

## Definitions of Networked Learning

Networked learning grew out of practices in open and distance learning, lifelong learning, computer-mediated communication, co-operative and collaborative learning, problem-oriented project pedagogy and critical and emancipatory pedagogy (Jones and Dirckinck-Holmfeld 2009; McConnell, Hodgson, and Dirckinck-Holmfeld 2012; Goodyear 2014; Hodgson and McConnell 2019). The links to co-operative and collaborative learning are clear in McConnell’s early formulation.

Networked collaborative learning (NCL) is therefore the bringing together of learners via personal computers linked to the Internet, with a focus on them working as a ‘learning community’, sharing resources, knowledge, experience and responsibility through reciprocal collaborative learning. (McConnell 1998)

Revisiting the literature of networked learning, over the last 20 years or so, reveals the breadth of its intellectual foundations (see Table 1).

**Table 1:** Networked learning: intellectual foundations

Socio-cultural accounts of learning and change, social constructivism, activity theory, and expansive learning	Vygotsky, Engeström
Critical pedagogy	Freire, Giroux, McLaren, hooks, Negt
Democratic education and experiential learning	Dewey, Kolb
Deschooling, learning webs, and convivial tools	Illich, Alexander
Adult learning, workplace learning, and professional development	Knowles, Lindeman, Brookfield, Schön
Humanistic psychology and student-centred learning	Rogers
Action research and action learning	Lewin, Revans, Kemmis
The German–Continental pedagogical tradition	Humboldt, Klafki, Negt
Problem-oriented project pedagogy (POPP)	Illeris, Negt, Dewey, Freire
Computer-mediated communications (CMC), online discussions	Hiltz, Harasim
Co-operative and collaborative learning; Computer-supported collaborative learning (CSCL)	Johnson and Johnson; Dillenbourg, O’Malley, Koschmann
Collaborative knowledge building in (distributed) communities of practice	Lave, Wenger, Scardamalia, Bereiter, Paavola
Science and Technology Studies (STS), socio-materiality and Actor-Network Theory	Orlikowski, Winch, Ingold, Fenwick, Latour, Law
Sociological and mathematical analyses of networks	Castells, Granovetter, Barabási, Wellman
Feminism and feminist poststructuralism	Gore, Ellsworth
Posthumanist and postdigital theory	Hayles, Haraway, Cascone
Human capability approach	Nussbaum, Sen

Networked learning crystallized in the late 1990s by distinguishing itself from developments in digital education that were undermining human connectivity—developments that threatened to reduce education to the production, delivery and consumption of ‘content’ (‘online materials’). What has become the customary definition of networked learning emphasizes inter-personal connections. It runs as follows:

We define ‘networked learning’ as learning in which [information and communications technologies are] used to promote *connections*: between one learner and other learners, between learners and tutors; between a learning community and its learning resources. Some of the richest examples of networked learning involve interaction with on-line materials *and* with other people. But use of on-line materials is not a *sufficient* characteristic to define networked learning. (Goodyear, Hodgson, and Steeples 1998: 2, original emphasis; or Goodyear, Banks, Hodgson, and McConnell 2004: 1-2).

Missing from this definition is any explanation of what the connections are *for*—it is silent about activity and purpose. Word choices within the definition also suggest that networked learning is restricted to formal education—in which people have defined roles (as learners and tutors) and in which learning is intentional (rather than incidental). This omission and circumscription are serious deficiencies.

In the last 20 years, some authors have tried improving upon the definition or have argued that networked learning has little intrinsic coherence (Hansen 2018). While some might say it is sustained only by the work of people who identify with the term, others have sought out more constructive interpretations.

For example, Dohn, Sime, Cranmer, Ryberg, and De Laat (2018) and De Laat and Dohn (2019) have identified four understandings of networked learning, each of which places emphasis on a different set of connections.

1. An emphasis on connections between people and how they develop, maintain and learn from networks of others.
2. An emphasis on connections between situations or contexts—how people make connections between such situations, transforming or reconstructing knowledge for use in different situations.
3. An emphasis on the ICT infrastructure and how it enables connections across time and space, including connections between situations (as in No. 2 above), boundary crossing, mobility, etc.
4. An emphasis on connections between (human and non-human) actants – understanding learning situations as entanglements of people and things.

While working broadly within the frame of the customary definition, Jones has this to say about purposes:

Networked learning has always been interested in equipping people with the capacity to work creatively, to identify and construct problems to work on, to find the resources to deal with the problems identified and to develop workable solutions. This approach is built with flexibility in mind. It is not tied to the details of a curriculum or a testing regime that measures specified outputs, but it builds resilience and the capacity to deal with change. (Jones 2015: 241)

Ponti and Hodgson (2006) developed, and Hodgson and McConnell (2019) recap, eight principles underpinning networked learning designs/programs:

1. The focus is on learning which has a perceived value to the learners.
2. Responsibility for the learning process should be shared (between all actors in the network).
3. Time has to be allowed to build relationships.
4. Learning is situated and context dependent.
5. Learning is supported by collaborative or group settings.
6. Dialogue and social interaction support the co-construction of knowledge, identity and learning.
7. Critical reflexivity is an important part of the learning process and knowing.
8. The role of the facilitator/animator is important in networked learning.

Critical and emancipatory dispositions have been a strong undercurrent in networked learning, though they do not always surface in summaries of the field. One might also argue that criticism has often been reserved for technological evangelism and the predatory commercial behaviours of players in the educational technology industry. This critical stance is a useful corrective to technological determinism (Jones 2015; Hodgson and McConnell 2019). It can help provide a brake on education’s susceptibility to fads, fashions and quick fixes. But a focus on the promoters and sellers of technology leaves many stones unturned, as even a brief reflection on the absences in Table 1 reveals. Scanning through the papers presented at networked learning conferences and through chapters in the corpus of networked learning books, one finds very little—not nothing, but surprisingly little—on such areas as critical race studies, postcolonialism, indigenous knowledge, class, gender studies, queer theory, disability studies, green and blue environmentalism and sustainability. Contributions and theory from disadvantaged spaces and the Global South are few and far between.

Critical and emancipatory dispositions appear in weaker and stronger forms. Or perhaps it would be more helpful to say that they sometimes feature in accounts of inquiry and action that are tightly bound to the pragmatics of local organizational contexts. Good examples include instances of networked action research and professional development through action learning. And they sometimes feature in much deeper and/or wide-ranging critiques of the structures and circumstances in which (networked) learning takes place (see, e.g. Jandrić and Boras 2015; Ryberg and Sinclair 2016; Littlejohn, Jaldemark, Vrieling, and Nijland 2019).

In revising our description of networked learning, this interest in forms of emancipatory action research, underpinned by a commitment to social justice and empowerment, needs to find a place. This also implies that we should situate a revised definition within larger action-oriented projects and/or promote its application in broader educational, social and political movements (Jones 2019).

The Covid-19 pandemic, the lockdowns, the loss of jobs and shrinking of economies (as classically measured) combine to make a time for reconsidering the shapes to be taken by a ‘new normal’ (Peters et al. 2020). The pandemic may have temporarily shifted global attention away from the growing perils of climate change. But the only ‘new normal’ worth fighting for has to be constructed within the constraints of environmental sustainability and has to address poverty, inequality and other forms of injustice (Raworth 2017). As Manzini (2005, 2015), Cottam (2019) and others have argued, finding just transitions to more sustainable ways of living necessarily involves collaborative forms of inquiry, design, social innovation and activism, underpinned by stronger, trusting human relationships.

This has at least two sets of implications for networked learning. The first is that enrolling students in networked learning practices should help them build the capabilities they need to participate in searching for and constructing better ways of living. The second is that enrolling more teachers in networked learning practices should help transform the character of our educational institutions: bringing to life what Kathleen Fitzpatrick has described as ‘generous thinking’, strengthening connections between institutions and communities and helping realize what Ronald Barnett dubbed the ‘ecological university’ and what Raewyn Connell simply calls ‘The Good University’ (Fitzpatrick 2019; Nørgård, Mor, and Bengtsen 2019; Barnett 2018; Connell 2019).

## Towards a Revised Definition of Networked Learning

There are five constituent parts to our conception of networked learning. Firstly, it involves processes of collaboration. To make this more comprehensive, we also invoke ideas of co-operation and collective action. Secondly, it involves processes of ‘coming to know’ and of acting on the implications of that knowledge. This does not privilege any one form of knowledge or learning. Thirdly, these processes depend on human relationships: they require and strengthen trust and reciprocity. Fourthly, a network’s activities have a larger purpose: they matter to the people involved. For example, they may enable participants in the network to see and act upon opportunities for valued change. Finally, there is the matter of enabling technologies. The customary definition made the use of information and communication technologies (ICTs) an integral part of networked learning, implying but not explicitly requiring that these be digital. A postdigital lens quickly reveals the damage done by simple oppositions: framing the world as digital or material, virtual or real, online or face-to-face, artificial or natural, technical or human. So the fifth and final element of our conception of networked learning offers a chance to go back, rescue and revive a term from our deeper past: Ivan Illich’s notion of ‘tools for conviviality’.

We must come to admit that only within limits can machines take the place of slaves; beyond these limits they lead to a new kind of serfdom. Only within limits can education fit people into a man-made environment: beyond these limits lies the universal schoolhouse, hospital ward, or prison ... Once these limits are recognized, it becomes possible to articulate the triadic relationship between persons, tools, and a new collectivity. Such a society, in which modern technologies serve politically interrelated individuals rather than managers, I will call ‘convivial’. (Illich 1973)

Convivial tools are those which lend themselves to creative use by networks of people who are joined in one or more shared social or political projects. They afford opportunities for people to make their lives together (*con* ‘with’ + *vivere* ‘live’).

Pulling these five elements together, we can say that

Networked learning involves processes of collaborative, co-operative and collective inquiry, knowledge-creation and knowledgeable action, underpinned by trusting relationships, motivated by a sense of shared challenge and enabled by convivial technologies.

By implication, human activities that share these characteristics can be defined as examples of networked learning.

To capture the points made earlier about the range and importance of connections, we also observe that

Networked learning promotes connections: between people, between sites of learning and action, between ideas, resources and solutions, across time, space and media.

## Situating the Use of a Definition

We identify three main kinds of situation in which a definitional text may be useful:

1. To enable researchers who are conducting a meta-analysis or systematic review to determine whether an intervention described in the literature is, or is not, an example of networked learning.
2. As a concise description of a field of research and practice—to be used in calls for contributions to networked learning conferences and publications.
3. To alert teachers, educational leaders and policy makers to the existence of this field of research and practice, as a robustly grounded alternative to ‘online’ or ‘blended’ learning, for example.

The first of these needs can be dealt with by decomposing a text, listing classificatory criteria and then determining whether an instance in the literature should or should not be classified as ‘networked learning’. Table 2 illustrates an approach, but we leave further work of this kind to others.

**Table 2:** Networked learning: deriving inclusion criteria for purposes such as systematic review^1^

Involves collaborative, co-operative, and/or collective learning activities?	Yes/No
Places special value on inter-personal relationships?	Yes/No
Learning activities based on a shared challenge?	Yes/No
Depends upon digital technologies?	Yes/No
Results in stronger connections between participants?	Yes/No
Results in generative and/or actionable ideas?	Yes/No

^1^We do not endorse this operational definition of networked learning. It is simply an example of how reductive approaches to research may function

The need for a concise description of networked learning, to be used in calls for conference papers and related publications, can be served by merging texts, as follows:

Networked learning involves processes of collaborative, co-operative and collective inquiry, knowledge-creation and knowledgeable action, underpinned by trusting relationships, motivated by a sense of shared challenge and enabled by convivial technologies. Networked learning promotes connections: between people, between sites of learning and action, between ideas, resources and solutions, across time, space and media.

Since conference organizers and book editors enjoy considerable freedom of action, we imagine that they will modify, contextualize and/or supplement this text as the need arises.

Finally, there is the matter of advocacy in the broader fields of educational policy and practice. A working description or definition of networked learning cannot do much on its own. Fortunately, at various points in the past, the networked learning community has made very productive use of *manifestos*—both to galvanize thinking and discussion (in their creation) and to represent the purposes and values of the field to others, promoting equality or de-centring the human, for example (Beaty, Hodgson, Mann, and McConnell 2002; Beaty, Cousin, and Hodgson 2010). Drawing on Latour (2010), Bayne and Ross (2016) have this to say about the nature of manifestos.

[W]e need to re-think the conventional purpose of the manifesto as an anti-reactionary revolutionary call-to-arms by an avant-garde committed to the ideal of progress. … The manifesto is contingent, open to debate, to change, to re-working as the field itself shifts: it is a ‘call to attention’ rather than a call to arms. (Bayne and Ross 2016: 127-8)

[A manifesto makes] explicit (that is, manifest) a subtle but radical transformation in the definition of what it means to progress, that is, to process forward and meet new prospects. Not as a war cry for an avant-garde to move even further and faster ahead, but rather as a warning, a call to attention, *so as to stop going further in the same way as before* toward the future. (Latour 2010: 473, emphasis added)

In other words, one use of ‘networked learning’ is to deflect a headlong march towards ever-cheaper, ever-poorer, ‘content-led’ manifestations of online education. This has been a recurring struggle whenever new technologies are positioned as a means to improve education and make it more efficient, glossing over an underlying policy or commercial motive linking automation and cost-reduction.

One can go further. Two illustrations will have to suffice. First, we might say that networked learning, so conceived, expresses an important point about connections between learning and change in the world. It resonates with these words:

[C]ollaborative purposeful transformation of the world is the core of human nature and the principled grounding for learning and development (Stetsenko 2008: 474)

[L]earning is not a process whereby stable, unchanging things become known by unchanging individuals. Rather, learning comprises changes in the conditions of human life and activity, in which both individuals and environments change … This is a change that involves not only the intellect but the whole person and how one relates to oneself and to others. Experience changes not only the way we intellectually know the world but also the way we affectively and perceptually relate to it (Damşa and Jornet 2016: 41)

Second, foregrounding learner agency, ‘expansive learning’, reflexivity and shared commitment to change inflects networked learning with a distinctive dynamic potential. Providing an infrastructure for shared critique, inquiry and the ongoing design of new tasks, technologies, resources and relationships starts a living process that is hard to stop.

## Points of Entry into the Networked Learning Literature

The monograph by Jones (2015) offers an excellent introduction to key ideas and issues in networked learning. Monographs by McConnell (1994/2000, 2006) have also been highly influential within the field.

Papers presented at the biennial networked learning conferences over the last 20 years are on open access.^1^ Revised versions of selected papers from many of the conferences have been gathered together in edited collections. Each of these books also includes introductions to, and/or reviews of, the state of the networked learning field, written by the volume editors. Selected papers from the five networked learning conferences held between 2010 and 2018 can be found in Dirckinck-Holmfeld, Hodgson, and McConnell (2012); Hodgson, De Laat, McConnell, and Ryberg (2014); Ryberg, Sinclair, Bayne, and De Laat (2016),; Dohn, Cranmer, Sime, De Laat, and Ryberg (2018); and Dohn, Jandrić, Ryberg, and De Laat (2020), respectively. Goodyear et al. (2004) offers selected papers from the 2002 conference.

The Hodgson et al. (2014) and later volumes appear in the Springer book series *Research in networked learning.*^2^ Hodgson and McConnell were the founding editors for this book series. Dohn, De Laat, and Ryberg took over editing the series in 2020. The *Research in networked learning* series also includes the Jones (2015) monograph and two edited collections focussing on critical perspectives in networked learning (Jandrić and Boras 2015) and networked professional learning (Littlejohn et al., 2019).

Five other books from outside the *Research in networked learning* series should also be mentioned. The collection edited by Steeples and Jones (2002) offers the first comprehensive overview of work in the field. Then there are four edited volumes that focus on the analysis of networked learning practices and architectures, and on design for networked learning: Dirckinck-Holmfeld, Jones, and Lindström (2009); Carvalho and Goodyear (2014); Carvalho, Goodyear, and De Laat (2017); and Dohn (2018).

## Concluding Comments

A core goal for this paper has been to open up discussion about the place of critical and emancipatory dispositions within current descriptions of networked learning. In proposing an updated characterization of the essence of networked learning, the paper suggests that greater attention needs to be paid to collective social projects that require both inquiry and action. Within this discussion, we must not forget to address a number of tensions within networked learning research and praxis—tensions that may become more severe if we are not alert to their nature and possible consequences. Three stand out. Firstly, there is the role of the teacher. Reflection on networked learning practices within formal education provides opportunities to consider aspects of the role of people who are in teaching positions. How teaching, facilitation, animation, leadership and network support roles should be considered in networks more broadly is a wide-open topic. So is the question of assessment. To what extent are conceptions of feedback, assessment and evaluative judgement appropriate in variously situated networks? Last, but not least, there are open questions about organizational and policy issues, which need deeper exploration as we find new spaces for networked learning.

## Footnotes

1 Past conference proceedings of networked learning conferences can be found at https://www.networkedlearning.aau.dk/past-conference-proceedings/. Accessed 25 June 2020.

2 See https://link.springer.com/bookseries/11810. Accessed 25 June 2020.
